# Case Report: Management Approach and Use of Extracorporeal Membrane Oxygenation for Diffuse Alveolar Hemorrhage After Pediatric Hematopoietic Cell Transplant

**DOI:** 10.3389/fped.2020.587601

**Published:** 2021-01-13

**Authors:** Kimberly Fan, Caitlin Hurley, Michael J. McNeil, Asya Agulnik, Sara Federico, Amr Qudeimat, Arun Saini, Jennifer McArthur, Ronald Ray Morrison, Hitesh Sandhu, Samir Shah, Saad Ghafoor

**Affiliations:** ^1^Division of Pediatric Critical Care, University of Tennessee Health Science Center, Memphis, TN, United States; ^2^Division of Critical Care, St. Jude Children's Research Hospital, Memphis, TN, United States; ^3^Department of Oncology, St. Jude Children's Research Hospital, Memphis, TN, United States; ^4^Division of Solid Tumor, Department of Oncology, St. Jude Children's Research Hospital, Memphis, TN, United States; ^5^Department of Bone Marrow Transplant, St. Jude Children's Research Hospital, Memphis, TN, United States; ^6^Division of Pediatric Critical Care, Texas Children's Hospital and Baylor College of Medicine, Houston, TX, United States

**Keywords:** diffuse alveolar hemorrhage (DAH), hematopoitic stem cell transplantation, extracorporeal mebrane oxygenation, extracorporeal life support (ECLS), pediatric acute respiratory distress syndrome

## Abstract

**Introduction:** Diffuse alveolar hemorrhage (DAH) is an early pulmonary complication of hematopoietic cell transplantation (HCT) associated with severe hypoxemic respiratory failure and mortality. Extracorporeal membrane oxygenation (ECMO) support is often used for respiratory failure refractory to conventional interventions; however, its use has been limited in HCT patients with DAH due to potential for worsening alveolar hemorrhage and reported high mortality.

**Case Presentation:** We report two cases of DAH following HCT who developed refractory hypoxemic respiratory failure despite cessation of bleeding and were successfully supported with ECMO.

**Conclusion:** DAH after HCT should not automatically preclude ECMO support; rather, these patients must be evaluated individually for ECMO within the context of their overall clinical picture.

## Introduction

Diffuse alveolar hemorrhage (DAH) is a known pulmonary complication following hematopoietic cell transplant (HCT). It usually occurs within the first 30 days following HCT and is diagnosed clinically by a constellation of hypoxemic respiratory failure, diffuse pulmonary infiltrates on chest radiography and progressively bloody bronchoalveolar lavage return on bronchoscopy (1–3). The pathogenesis of DAH is not clearly understood, but it is thought to develop from a combination of intrinsic lung injury, dysregulated inflammation, and cytokine release (1). It is classically considered a non-infectious process, although it may be precipitated by occult infections (3, 4). While systemic glucocorticoids are considered the mainstay of therapy, there is high variability in dosing, duration of therapy, and a reported mortality over 40% within the pediatric population (2, 3). More recently, cohorts of pediatric HCT patients treated with newer therapeutic agents, such as nebulized tranexamic acid (TXA) and intrapulmonary recombinant activated human factor VII (IP-rFVIIa), have demonstrated improved survival to over 65% (5, 6). Interventions such as extracorporeal membrane oxygenation (ECMO) have been used in non-HCT DAH (7, 8). HCT has historically been considered a contraindication for ECMO by some centers; therefore, its use has been limited (9). To our knowledge, there has only been one case report in the literature of ECMO use for DAH following HCT in pediatrics (10). We report two cases of pulmonary hemorrhage following HCT successfully supported with ECMO and present our approach to the management of these unique and complex patients.

## Case 1

### Case Presentation

An 18-year old female with Diamond-Blackfan anemia underwent a 10/10 matched unrelated donor (MUD) HCT, following a conditioning regimen consisting of cyclophosphamide, busulfan, fludarabine, thiotepa, and rabbit anti-thymoglobulin. The initial post-transplant course was complicated by fungal sepsis with *Candida parapsilosis* and a large right atrial thrombus for which she underwent open thrombectomy on cardiopulmonary bypass.

**Figure 1:**
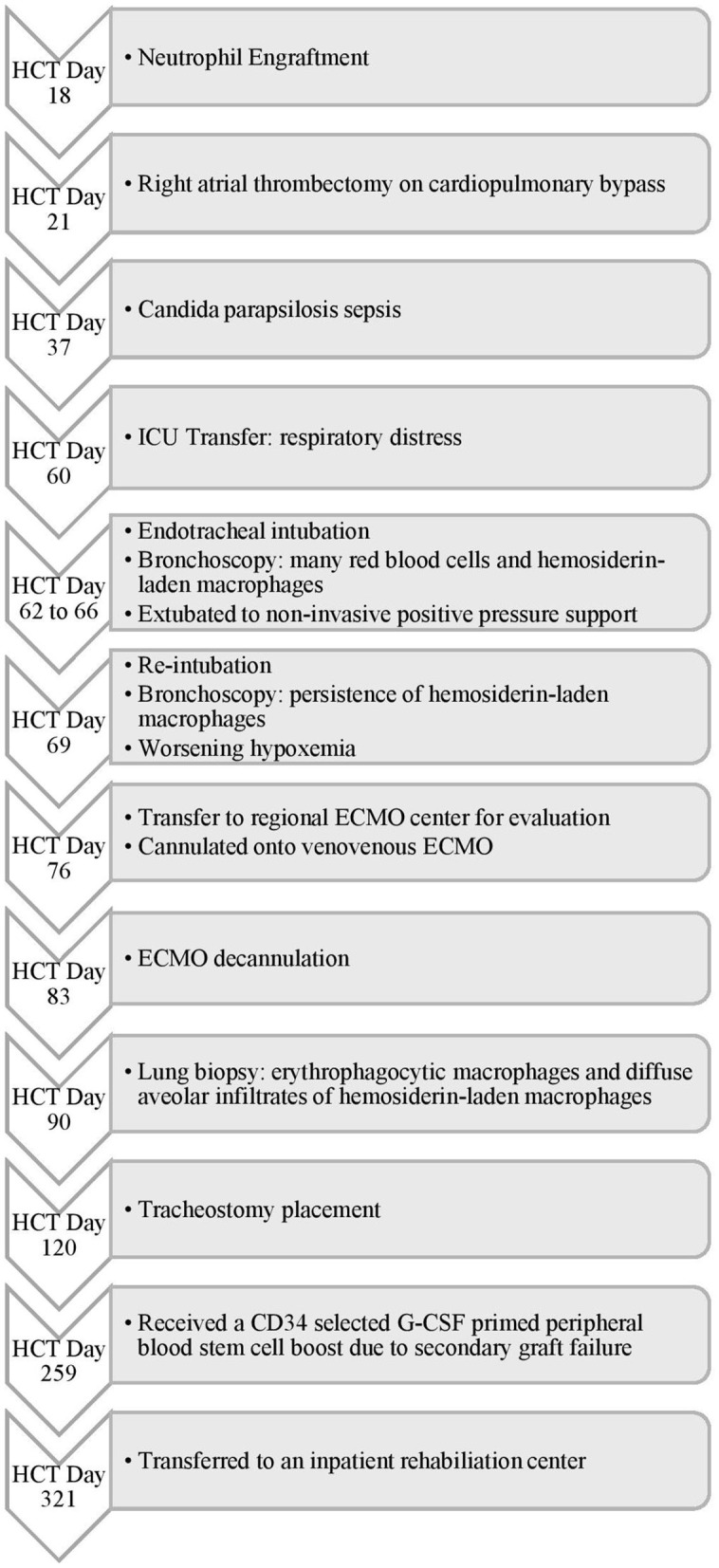
Timeline of Events for Case 1. HCT, hematopoietic cell transplant; ICU, intensive care unit; ECMO, extracorporeal membrane oxygenation; CD, cluster of differentiation; G-CSF, granulocyte-colony stimulating factor.

**Figure 2:**
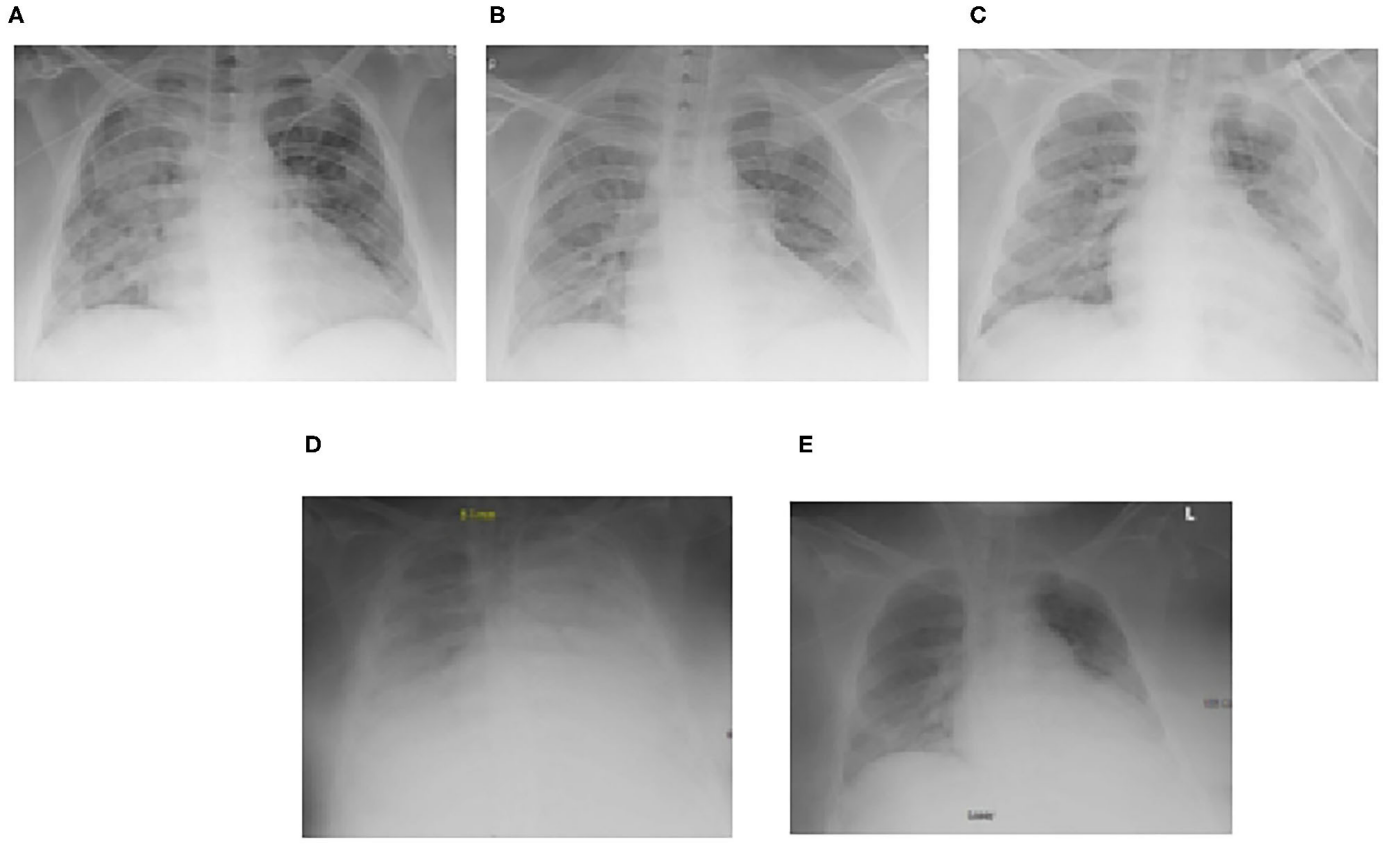
Serial chest radiographs for Case 1 demonstrating the progression and subsequent improvement of diffuse bilateral interstitial and alveolar opacities. **(A)** Initial decline prompting transfer to the ICU, **(B)** Mild respiratory improvement, **(C)** Worsening ARDS leading to transfer for ECMO evaluation, **(D)** ECMO–Cannulation, **(E)** Pre-decannulation.

On HCT day 60 (Figure 1), she developed respiratory distress with bilateral pulmonary infiltrates on chest radiography. She was transferred to the intensive care unit (ICU) with worsening hypoxemia progressing to endotracheal intubation and mechanical ventilation. Bronchoscopy demonstrated many red blood cells and hemosiderin-laden macrophages consistent with DAH but no active hemorrhage. Bronchoalveolar lavage revealed positive aspergillus antigen, for which she was continued on antifungal therapy for probable pulmonary aspergillosis. She improved with supportive management and was extubated to non-invasive positive pressure support. However, on HCT day 69, she developed new bilateral infiltrates on chest radiography requiring reintubation (Figure 2). A repeat bronchoscopy showed persistence of hemosiderin-laden macrophages without evidence of active bleeding.

By HCT day 76, she progressed to severe acute respiratory distress syndrome (ARDS) with a peak oxygenation index (OI) of 40. Given her acute deterioration, poor organ reserve, and high mortality risk, she was transferred to the regional pediatric ECMO program at Le Bonheur Children's Hospital and emergently cannulated onto Veno-Venous (VV) ECMO with placement of two-21 French Biomedicus (Medtronic, Minneapolis, MN, USA) cannulas in the right femoral and right internal jugular veins. She was adequately supported with initial flows of 2.16 liters per minute (LPM), ~40 ml/kg/min, and sweep gas flow of 3 LPM using the Maquet Rotaflow centrifugal pump and Quadrox-iD adult oxygenator (Maquet, Harlingen, Germany). She was anticoagulated on our institution's bleeding protocol with unfractionated heparin targeting an activated clotting time (ACT) of 180–200 s and transfused with packed red blood cells (PRBC) and platelets to keep hematocrit > 35% and platelet count > 75 × 10^9^/L, respectively. She was treated with intravenous methylprednisolone 2 mg/kg/day for 7 days followed by a 6 week wean. She was successfully decannulated after 7 days of VV ECMO support. There was no recurrence of pulmonary hemorrhage on ECMO and she was transferred back to the referring institution 3 days after decannulation.

After decannulation, she had multiple pulmonary hemorrhage episodes treated with methylprednisolone, aminocaproic acid, and IP-rFVIIa. A lung biopsy on HCT day 90 was indicative of recent and remote alveolar hemorrhages. Due to her recurrent pulmonary hemorrhages and evolving practice for early tracheostomy placement at that time, her tracheostomy was delayed for more than 60 days following her initial respiratory failure. She was considered a higher risk for early tracheostomy due to secondary graft insufficiency. She received a CD34+ cell boost on HCT day 259. After extensive inpatient rehabilitation, she was liberated from her tracheostomy and is successfully surviving 4 years after HCT.

## Case 2

### Case Presentation

**Figure 3:**
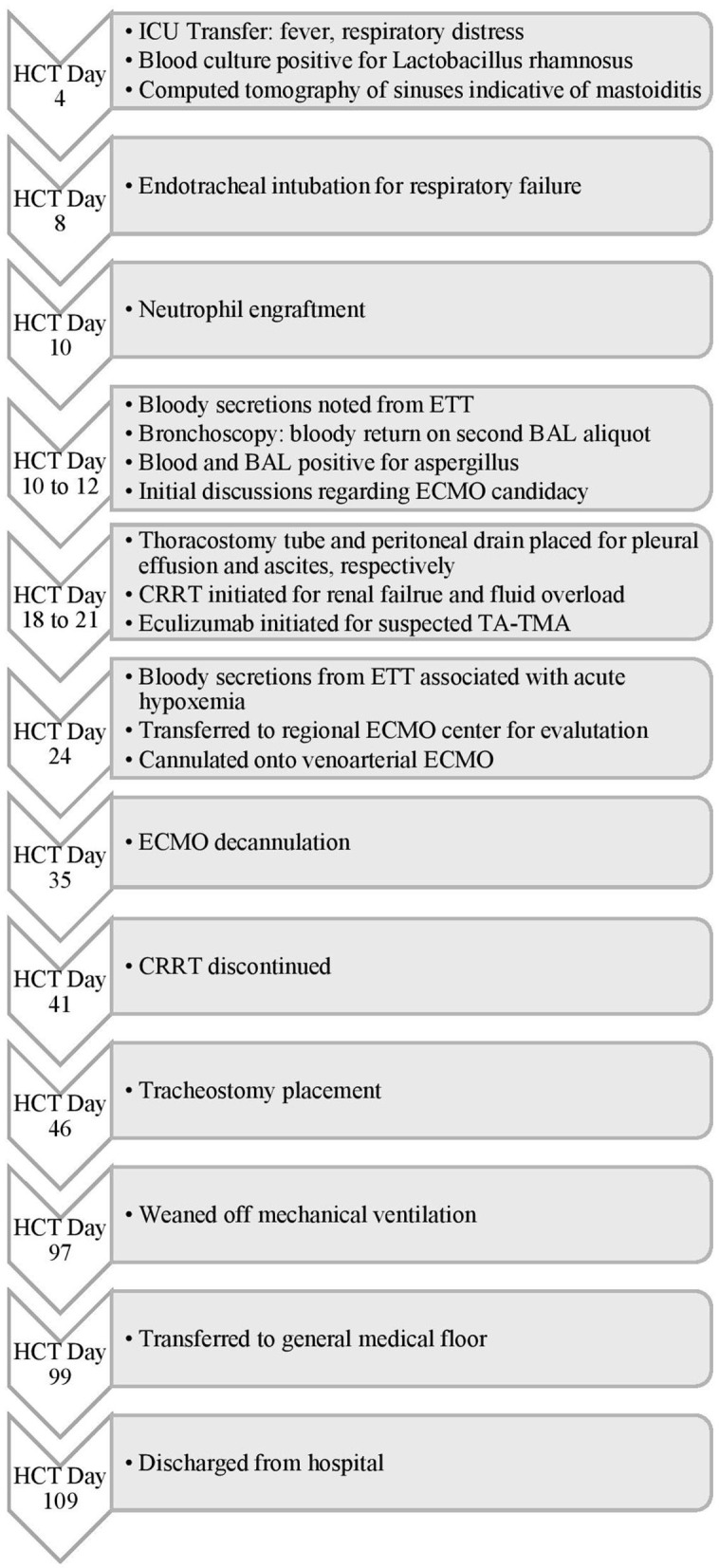
Timeline of Events for Case 2. HCT, hematopoietic cell transplant; ICU, intensive care unit; ETT, endotracheal tube; BAL, bronchoalveolar lavage; ECMO, extracorporeal membrane oxygenation; CRRT, continuous renal replacement therapy; TA-TMA, transplant-associated thrombotic microangiopathy.

A 4-year-old male with metastatic neuroblastoma underwent his second tandem autologous HCT following a conditioning regimen of melphalan, etoposide, and carboplatin. His medical history was notable for acute kidney injury secondary to cisplatin with non-functioning right kidney on imaging. His transplant course was complicated by fever and severe mucositis and mild sinusoidal obstructive syndrome (SOS). On HCT day 4 (Figure 3), he was transferred to the ICU with systemic inflammatory response and worsening respiratory distress and was found to have *Lactobacillus rhamnosus* sepsis and mastoiditis. Despite treatment with broad-spectrum antibiotics and methylprednisolone 1 mg/kg/day, he had worsening respiratory distress requiring endotracheal intubation on HCT day 6. He subsequently developed bloody secretions in his endotracheal tube (ETT) which led to the discontinuation of defibrotide. Bronchoscopy showed copious mucopurulent secretions in the right lower lobe which became bloody with the second aliquot on BAL and were positive for *Aspergillus*. He was therefore treated for presumed fungal pneumonia. Methylprednisolone 1 mg/kg/day for 4 days and nebulized TXA (n-TXA) 250 mg were used for pulmonary hemorrhage control. In addition, a mean airway pressure (MAP) > 15 cm H_2_O was targeted to tamponade the bleed. He was transfused to maintain a hemoglobin > 7 gram/dL and a platelet count over 100 × 10^9^/L, respectively. Hemostasis was achieved, however, he developed severe ARDS with a peak OI of 25. The ECMO liaison team initiated early joint discussions regarding ECMO candidacy at this time. The patient developed renal failure and fluid overload necessitating continuous renal replacement therapy (CRRT). Further, he had a rising lactate dehydrogenase, refractory hypertension and thrombocytopenia, he was treated with eculizumab for suspicion of transplant-associated thrombotic microangiopathy (TA-TMA).

**Figure 4:**
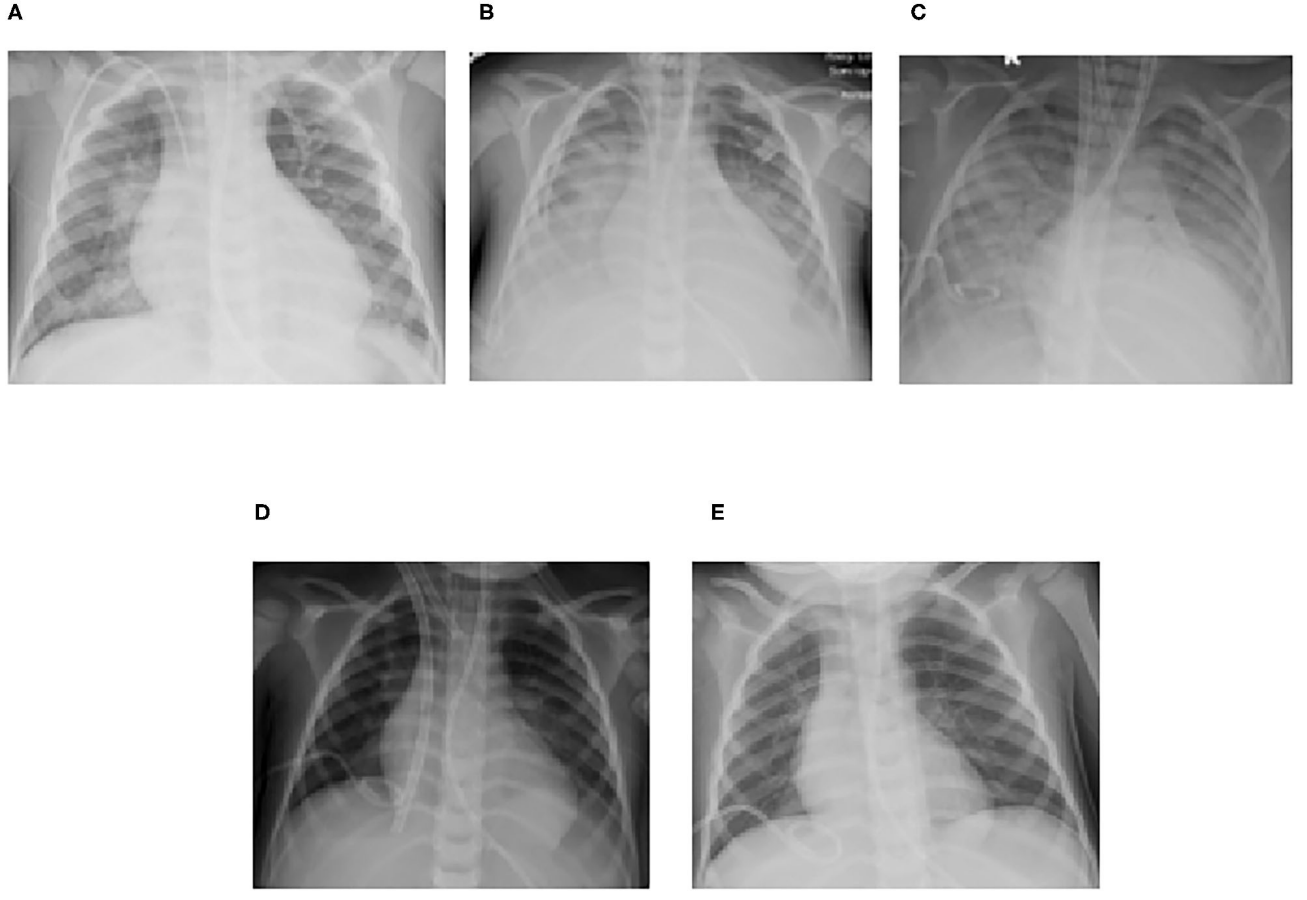
Serial chest radiographs for Case 2 demonstrating the progression and subsequent improvement of diffuse bilateral interstitial and alveolar opacities **(A)** Following intubation, **(B)** Prior to transfer for ECMO evaluation, **(C)** Following ECMO cannulation, **(D)** Prior to ECMO decannulation, **(E)** Post-transfer back to referring center.

On HCT day 24, he had an acute hypoxemic event with copious bloody secretions in his ETT. Chest radiography showed increased pulmonary opacities (Figure 4). Coagulation factors at that time included a platelet count of 97 × 10^9^/L, and normal PT, INR, aPTT and fibrinogen levels. He was treated with intravenous TXA 1,000 mg bolus followed by 10 mg/kg/hr infusion, nebulized TXA 250 mg, and 3 doses of intrapulmonary rFVIIa (IP-rFVIIa) 50 μg/kg. Despite achieving hemostasis following IP-rFVIIa and escalation of his respiratory support to HFOV, he had progressive hypoxemia with an OI of 68. All infectious studies were negative. The added risk of mortality with multiorgan failure and SOS were discussed between the clinical and ECMO liaison teams but he was considered a candidate for ECMO due to the reversibility of his organ dysfunction. Due to his clinical acuity and high risk of death on the current support, he was transferred to the regional ECMO program. Upon arrival at the referral center, he was placed on HFOV while awaiting cannulation. Due to his small size and the presence of a femoral vascath, he was cannulated onto VA ECMO with a 14 French arterial cannula in the right carotid artery and a 15 French venous canula in the right internal jugular vein (BioMedicus Medtronic, Minneapolis, MN, USA). He was supported with initial flows of 1.3 liters per minute (LPM), ~110 ml/kg/min, and sweep gas flow of 0.6 LPM using the Maquet CardioHelp centrifugal pump and HLS Set Advanced 7.0 Oxygenator (Maquet, Rastatt, Germany). For anticoagulation, he was placed on a bivalirudin infusion per institutional preference, titrated to maintain aPTT of 40–60 s. Given the need for systemic anticoagulation, he was treated with three additional doses of nebulized IP-rFVIIa 50 μg/kg and received IV methylprednisolone 2 mg/kg/day and n-TXA 250 mg every 6 h throughout his ECMO course. He had no further episodes of DAH or thromboembolic complications. CRRT was continued for fluid overload. He was maintained on higher MAP during the course of his ECMO stay. He was decannulated after 10 days of ECMO support and transferred back to the referring institution the following day.

Following return of renal function, CRRT was discontinued. He underwent tracheostomy placement 11 days following ECMO decannulation to allow gradual weaning of mean airway pressure and facilitate rehabilitation. He was weaned off mechanical ventilation support, transferred to the general medical floor under the care of the oncology team for continued radiation and chemotherapy, and successfully discharged to outpatient care on HCT day 109. He remains well 6 months following hospital discharge.

## Family Perspectives

We asked the families to share their thoughts and experiences through the course of their children's illness and hospital course. As expected, the families' found the process stressful but were ultimately relieved and grateful for the outcome.

### Perspective From the Mother of Patient 1

“There are things I remember so vividly about my daughter's experience on ECMO, and things that I think my mind has chosen to forget out of self-protection. I remember listening through the glass doors in the ICU, [the intensivist] arguing for why my daughter should be [an ECMO candidate]. I remember watching the team wheel her out [for transfer to the ECMO referral center], and the look on their faces is what sticks with me the most. They were trying so hard to be brave for us, but everyone knew […] that she may not come back. I was simply in survival mode at that point. My daughter doesn't remember much about ECMO, which is a blessing considering how difficult the experience was, both clinically and emotionally. I am forever grateful to everyone that played a part in saving my daughter's life, and my sanity during such trying times.”

### Perspective From the Mother of Patient 2

When describing the experience, our patient's mother's first response was that of shock. It was difficult for her to recall specific emotions or thoughts during this distressing experience. She expressed appreciation for the support she received from the medical team. She was particularly grateful for the careful and thoughtful approach from team members when making difficult medical decisions. She recognized it was not one individual deciding her son's care but a collaborative expert medical team. One statement she felt was unhelpful but heard repeatedly was “things will get worse before they get better.” She knew it was valuable information but hearing it continually became exasperating. She understood that once her son was on ECMO the only thing worse would be losing him. She is extremely grateful that he is better and doing well-today.

## Discussion

### Diagnosis and Evaluation

DAH is a known pulmonary complication of HCT with a historically high mortality rate (2, 3). While there is no standardized laboratory or radiographic feature pathognomonic for DAH, it is clinically diagnosed by respiratory failure, diffuse infiltrates on chest radiography, and restrictive lung disease. It is confirmed with progressively bloodier BAL on bronchoscopy or ≥20% hemosiderin-laden alveolar macrophages (1). The use of newer, more specific agents, such as n-TXA and IP-rFVIIa (5, 6), have improved survival over the last decade, DAH still carries significant morbidity and mortality. Therefore, we recommend a holistic, multidisciplinary team approach. At our institution, treatment of DAH is guided by the principles of early recognition and diagnosis, achieving hemostasis, decreasing inflammation, and ultimately allowing for endothelial healing. Post-HCT, patients are monitored closely in the transplant unit for changes in respiratory status or new findings on chest radiography. If these are detected, prompt bronchoscopy is performed to identify and treat potential infectious sources. As most patients with DAH eventually develop respiratory failure and require intensive care (2, 3, 11), patients are transferred to the ICU early for continued monitoring if DAH is suspected. Additional evaluation including computed tomography of the chest and early lung biopsy is considered. Our management plan rests on the hypothesis that endothelial damage underlies many complications of HCT (12). The diagnosis of TA-TMA is pursued early and eculizumab given when indicated. We provide supportive treatment to allow time for the endothelium to heal.

### Active Bleeding

Once DAH is diagnosed, our standardized treatment protocol includes systemic glucocorticoids and nebulized TXA, with the addition of IP-rFVIIa if there is refractory hemorrhage. Initially, methylprednisolone is dosed at 2 mg/kg/day with an accelerated taper over 4–8 weeks, particularly if there is concern for disseminated viral infection. We strongly consider the use of pulse dose glucocorticoids, methylprednisolone 15–30 mg/kg/day or dexamethasone 4-5 mg/kg/day, for 2–3 days followed by a taper over 4–8 weeks to maximize the genomic and non-genomic anti-inflammatory effects of glucocorticoids (13, 14). Nebulized TXA is given every 6 h over the first 18–24 h. If the bleeding continues, then IP-rFVIIa 50 μg/kg is added. IP-rFVIIa is initially administered every 15–30 min until hemostasis is achieved and subsequently continued every 4–6 h for a maximum of 3 days. Respiratory failure is managed with early endotracheal intubation and mechanical ventilation with MAP > 15 cmH_2_O to tamponade the bleeding. Continuous renal replacement therapy is considered early to manage fluid overload. During active bleeding, PRBC and platelet transfusions are used to maintain hemoglobin over 7 gram/dl and a platelet count over 50 × 10^9^/L respectively. Rotational thromboelastometry (ROTEM) is used to further guide therapy.

DAH has been described in the setting of TA-TMA (15), a subset of thrombotic microangiopathies characterized by the presence of schistocytes, elevated serum LDH, renal, and neurologic dysfunction (16). In the setting of TA-TMA, DAH has a reported mortality rate of up to 100% (15). Eculizumab is a complement inhibitor that is promising in the treatment of TA-TMA (17). Therefore, if there is suspicion of TA-TMA as in our second case, the addition of eculizumab is considered in collaboration with the transplant team. As occult infections have later been identified in patients with DAH despite initial negative cultures, we empirically start patients on broad spectrum antimicrobials and continue treatment for at least 48 h. A second bronchoscopy is frequently performed 2 to 5 days after the development of symptoms for repeat cultures.

Idiopathic pneumonia syndrome (IPS) is another non-infectious pulmonary complication of HCT and shares many clinical features with DAH. It is characterized by elevated levels of specific cytokines, including tumor necrosis factor receptor-1 (TNFR1), a marker for tumor necrosis factor- alpha (TNF-α). IPS is treated with glucocorticoids and etanercept, a TNF-α binding protein (18). Thus, if no infectious process is identified and IPS is of concern, treatment with etanercept is considered. For refractory hypoxemic respiratory failure despite cessation of bleeding, use of ECMO is considered as discussed below.

### Recurrent Bleeding

In cases of recurrent bleeding, in addition to the above treatments, we consider early tracheostomy with the goals of maintaining a high mean airway pressure with mechanical ventilation for >4 weeks while endothelial healing occurs and allowing for early mobilization and rehabilitation.

### ECMO Decision

The medical complexity of HCT care inherently raises the PICU mortality when compared to the general population (19). As such, underlying malignancy and HCT are considered contraindications for ECMO support by most centers. In a review of the Extracorporeal Life Support Organization (ELSO) database, Gow et al. identified 107 patients with underlying malignancies supported with ECMO from 1994 to 2007. Of these, 42% survived to ECMO decannulation and 35% survived to hospital discharge, suggesting that ECMO use may be a reasonable support modality in this patient population (20). Unfortunately, ECMO outcomes in HCT patients have been worse. In another review of the ELSO database from 1991 to 2004, Gow et al. reported a 21% and 5% survival to ECMO decannulation and hospital discharge, respectively, in HCT patients (9). In 2014, DiNardo et al. published an extended review of ECMO support post-HCT from the ELSO database from 1991 to 2012 and found that 21% survived to decannulation and 10% survived to hospital discharge (21). However, since these publications, there have been significant advancements in HCT care, including conditioning regimens, donor cell source selection, HLA matching, preparation and supportive care (22). Likewise, technological advances in ECMO support and improvements in the management of mechanical ventilated patients have resulted in improved ECMO outcomes (23, 24). Zinter et al. found a 22% PICU survival in HCT patients supported with ECMO from 2009 to 2014 (19). Most recently, in a review of HCT patients supported with ECMO from 2011 to 2018, Steppan et al. found that 4 out of 8 patients survived to hospital discharge (25). Additionally, successful ECMO after HCT cases not included in aforementioned reviews have been reported. Williams et al. reported a 18 month old patient with acute respiratory failure from rhinovirus infection and aspiration pneumonia after autologous HCT for neuroblastoma who was successfully supported with VV ECMO (26). Potratz et al. reported a 14 year old allogeneic HCT patient with acute respiratory failure secondary to engraftment syndrome successfully supported on VV ECMO (27). Morris et al. reported the successful use of ECMO for diffuse alveolar hemorrhage post-HCT in a single pediatric patient (10). Together with our patients, there are now 3 pediatric patients successfully supported for post-HCT DAH with ECMO support.

Choosing which HCT patient has a reasonable chance of recovery with ECMO support is challenging. In collaboration with our regional pediatric ECMO program at Le Bonheur Children's Hospital, we have developed a liaison team to assess ECMO candidacy with the goals of arranging timely transfer for ECMO evaluation and early ECMO initiation to limit significant ventilator-induced lung injury. This ECMO liaison team was instrumental in the process of case discussion and patient advocacy for transfer and early initiation of extracorporeal therapy in the cases reported here. The liaison teams consist of intensivists at both institutions and garners input from oncologists, HCT physicians and other specialties as needed on a case by case basis. We also review the indications, risks, and benefits of ECMO with the patient's family to allow them to make an informed decision regarding transfer for ECMO evaluation during these initial discussions. We begin ECMO candidacy discussions early—ideally days before the patient requires transfer for ECMO evaluation. In general, the criteria for ECMO initiation is similar to that which is used for our general pediatric population, with a few considerations specific to oncologic and HCT patients. HCT patients represent a heterogenous population with various underlying diseases, conditioning regimens, cell sources and post HCT comorbidities. These patients must be individually evaluated for the potential of ECMO benefit. The ECMO liaison team is invaluable for teasing out the nuances of individual patient factors.

Our report is limited by an inherent selection bias for HCT ECMO survivors who had cessation of pulmonary hemorrhage prior to ECMO initiation since active bleeding not controlled with medical management is considered an institutional contraindication for ECMO at this time. As such, we do not include patients with DAH who were not considered ECMO candidates in this case series. A concern in the HCT population has been that while ECMO supports the lungs, it may worsen other organ dysfunction and increase infection risk. This concern has limited the use of ECMO in the HCT population. Our cases highlight that HCT and DAH should not absolutely preclude patients from ECMO candidacy. HCT patients should be evaluated within the context of their overall clinical picture.

## Conclusion

ECMO support has historically been considered contraindicated in HCT patients given the complexity of their disease. Through the development of a multidisciplinary approach in treatment of DAH post-HCT, as well as an expert ECMO liaison team to evaluate ECMO candidacy, we successfully supported two patients with respiratory failure secondary to DAH refractory with ECMO. Over the last decade, there have been significant advances made in ECMO technology as well as the field of HCT, we argue that ECMO use should be considered in select HCT patients.

## Data Availability Statement

The original contributions presented in the study are included in the article/supplementary material, further inquiries can be directed to the corresponding author/s.

## Ethics Statement

Written informed consent was obtained from the individual(s), and minor(s)' legal guardian/next of kin, for the publication of any potentially identifiable images or data included in this article.

## Author Contributions

KF provided substantial contribution to the conception, literature search, and drafting of the manuscript. CH provided substantial contribution to the drafting and critical revisions of the manuscript. MM and SF obtained informed consent for publication of the second case, contributed to writing the family perspective, and provided critical revisions of the content. AA, AQ, AS, JM, RM, HS, and SS provided critical intellectual revisions of the manuscript content. SG provided substantial contribution to the conception, literature search, intellectual content, critical revision, approval of the final version of the manuscript, and agrees to be accountable for all aspects of the work related to accuracy and integrity. All authors contributed to the article and approved the submitted version.

## Conflict of Interest

The authors declare that the research was conducted in the absence of any commercial or financial relationships that could be construed as a potential conflict of interest.
